# Visual hallucinations in Parkinson’s disease are associated with thinning of the inner retina

**DOI:** 10.1038/s41598-020-77833-1

**Published:** 2020-12-03

**Authors:** F. Visser, V. I. Apostolov, A. M. M. Vlaar, J. W. R. Twisk, H. C. Weinstein, H. W. Berendse

**Affiliations:** 1 Department of Neurology, OLVG, Jan Tooropstraat 164, 1061 AE Amsterdam, The Netherlands; 2 Department of Ophthalmology, OLVG, Jan Tooropstraat 164, 1061 AE Amsterdam, The Netherlands; 3 Department of Epidemiology and Data Science, Amsterdam UMC, De Boelelaan 1089a, 1081 HV Amsterdam, The Netherlands; 4 Department of Neurology, Amsterdam UMC, Vrije Universiteit Amsterdam, Amsterdam Neuroscience, De Boelelaan 1117, 1081 HV Amsterdam, The Netherlands

**Keywords:** Neuroscience, Anatomy, Biomarkers, Medical research, Neurology, Pathogenesis, Signs and symptoms

## Abstract

Visual hallucinations (VH) are common in patients with Parkinson’s disease (PD), yet the underlying pathophysiological mechanisms are still unclear. We aimed to explore the association of the presence of VH with inner retinal thinning and, secondarily, with visual acuity. To this end, we included 40 PD patients in this exploratory study, of whom 14 had VH, and 22 age- and sex-matched healthy controls. All participants were interviewed for the presence of VH by a neurologist specialized in movement disorders and underwent a thorough ophthalmologic examination, including measurement of the best-corrected visual acuity (BCVA) and optical coherence tomography to obtain macular scans of the combined ganglion cell layer and inner plexiform layer (GCL-IPL). Patients with VH had a thinner GCL-IPL than patients without VH, which persisted after correction for age, disease stage, levodopa equivalent daily dose (LED) and cognitive function. Furthermore, BCVA was lower in the PD group with VH than in the PD group without VH, although only a trend remained after correction for age, disease stage, LED and cognitive function. Taken together, in patients with PD, visual hallucinations appear to be associated with a thinning of the inner retinal layers and, possibly, with reduced visual acuity. Further research using a longitudinal design is necessary to confirm these findings and to establish the causality of these relationships.

## Introduction

Visual hallucinations (VH) are common in patients with Parkinson’s disease (PD)^1–3^, yet the underlying pathophysiological mechanisms are still unclear. Over the course of the disease, minor hallucinations may first evolve into VH with retained insight and, subsequently, into multimodality hallucinations with loss of insight and delusions, corresponding to the development of the disease process from the brainstem, through the basal forebrain and ultimately involving widespread cortical brain areas^4^. According to the leading theory in the field, dysfunction of attentional networks in combination with ambiguous visual input may lead to VH when remembered images intrude into consciousness^5,6^. In support of this theory, ocular disorders have been associated with the presence of VH in PD^7^.

In PD, retinal involvement has been demonstrated in histopathological research, showing retinal dopamine deficiency as well as deposition of abnormal alpha synuclein in the inner retinal layers, more specifically, in the ganglion cell layer (GCL), the inner plexiform layer (IPL) and the inner nuclear layer (INL)^8–11^. These pathological changes may cause ganglion cell loss and, hence, GCL thinning, which has been demonstrated in vivo in PD patients^12^. Since the axons of the ganglion cells form the retinal nerve fibre layer (RNFL), ganglion cell loss may subsequently result in RNFL thinning^13,14^. Although RNFL thinning has been associated with the occurrence of VH^15^, this observation has been contradicted by others^16^. In a very recent study, that included all retinal layers, the combined GCL-IPL correlated best with reduced low contrast visual acuity and visual cognition outcomes in Lewy body disease^17^. Whether thinning of the GCL-IPL is associated with the occurrence of VH is currently unknown. We therefore aimed to explore the association of the presence of VH with GCL-IPL thinning and, secondarily, with visual acuity in PD.

## Results

### Patients

Clinical and demographic data of the included participants are presented in Table 1. Between the 40 PD patients and 22 controls, there were no significant differences in age, sex or comorbidity. Patients had a lower MoCA score than controls, as well as a lower intraocular pressure.

**Table 1:** Clinical characteristics and ophthalmologic data in controls and patient groups.

	HC n = 22	PD n = 40	PD+ n = 14	PD− n = 26
Age (years), median (IQR)	70 (14)	71 (13)	76 (13)	69 (13)
Male, n	12	28	11	17
Hypertension, n	4	9	4	5
Diabetes mellitus, n	2	5	2	3
MoCA score, median (IQR)	28 (2)	23 (4)	21 (10)	24 (4)
CLOX1 score, median (IQR)	14 (2)	12 (7)	9 (9)	13 (3)
MDS-UPDRS part III, median (IQR)	NA	22 (13)	27 (15)	19 (13)
Modified H&Y stage, median (IQR)	NA	2.5 (1)	2.5 (1.5)	2.0 (0.5)
Disease duration (years), median (IQR)	NA	7 (5)	7 (5)	7 (5)
Daily LED, median (IQR)	NA	675 (710)	750 (683)	675 (678)
On dopamine agonists, n	0	14	2	12
On psychotogenic medication, n*	0	5	2	3
Visual hallucinations, n	0	14	14	0
BCVA, median (IQR)	1.1 (0.3)	1.0 (0.3)	0.7 (0.8)	1.0 (0.3)
IOP ODS, median (IQR)	15 (4)	12 (3)	12 (4)	12 (3)
**Averaged thickness GCL-IPL**				
Temporal Q (µm), median (IQR)	69 (10)	67 (9)	62 (8)	70 (10)
Inferior Q (µm), median (IQR)	64 (9)	64 (9)	60 (8)	66 (7)
Nasal Q (µm), median (IQR)	69 (10)	68 (9)	65 (8)	70 (8)
Upper Q (µm), median (IQR)	67 (8)	66 (10)	60 (5)	67 (7)

*HC* healthy controls, *PD* patients with Parkinson’s disease, *PD* + patients with Parkinson’s disease with visual hallucinations, *PD*− patients with Parkinson’s disease without visual hallucinations, *IQR* interquartile range, *n* = number, *MoCA* Montreal Cognitive Assessment, *MDS-UPDRS part III* Movement Disorder Society—Unified Parkinson's Disease Rating Scale part III: motor section, *LED* Levodopa equivalent dose, *BCVA* best-corrected visual acuity, *IOP* intraocular pressure, *ODS* oculus dexter et sinister, *GCL-IPL* combined ganglion cell layer and inner plexiform layer, *Q* quadrant, *NA* not applicable.

*Medication with hallucinogenic side effects, including amantadine, anticholinergics and opioids.

Thirty-five percent of patients (n = 14) had VH, of whom three had minor hallucinations only (presence or passage hallucinations, or visual illusions), while the remainder of patients had complex visual hallucinations (seeing small animals, e.g. rats, or persons) or a combination of minor and complex hallucinations (with loss of insight in only two patients). VH occurred daily in 50%, weekly in 25% and monthly in 25% of the patients with VH. The 14 patients with VH were older than the patients without VH, see Table 1. The group with VH also had a lower MoCA score and CLOX1 score, and a higher MDS-UPDRS motor score, mHY stage and LED than the patient group without VH.

### Visual hallucinations and ocular measurements

We found no significant differences in GCL-IPL measurements between the PD group and the control group, yet visual acuity (BCVA) was lower in PD patients (median (IQR): 1.0 (0.3)) than in controls (median (IQR) = 1.1 (0.3), p < 0.01). In the PD group, the presence of VH was significantly associated with thinning of the GCL-IPL, see Table 2. Adjustment for age, levodopa dosage (LED), disease stage (mHY) and cognitive function (MoCA score) slightly increased the odds ratio, and the relationship remained statistically significant. However, after adjustment for age, LED, disease stage and cognitive function, only a trend remained for the association between VH and BCVA.

**Table 2:** Results of the logistic GEE analyses regarding the relationships between GCL-IPL thinning, BCVA and VH in PD.

	Crude			Adjusted^a^		
	OR	95% CI	p-value	OR	95% CI	p-value
VH vs GCL-IPL	1.18	1.07 – 1.30	0.001*	1.32	1.12 – 1.55	0.001*
VH vs BCVA^b^	1.48	1.14 – 1.90	0.003*	1.50	0.98 – 2.29	0.065

*GCL-IPL* combined ganglion cell layer and inner plexiform layer, *BC*VA best-corrected visual acuity, *VH* visual hallucinations, *vs* versus, *OR* odds ratio, *CI* confidence interval.

*A p value p < 0.05 was considered statistically significant.

^a^Adjusted for age, disease stage (modified Hoehn and Yahr stage), levodopa equivalent dose (LED) and cognitive function score (Montreal cognitive assessment—MoCA score).

^b^Expressed for a change of 0.1 unit in BCVA.

After correction for age, we did not find an association between GCL-IPL thinning and reduced BCVA, see Table 3. However, GCL-IPL thickness was correlated with global cognitive function (MoCA score). Furthermore, Table 3 illustrates that disease stage (mHY) was inversely correlated with GCL-IPL thickness (although only at trend level) and with BCVA. Lastly, disease stage was associated with the presence of VH.

**Table 3:** Correlations of GCL-IPL thickness with visual acuity and cognitive function, and of GCL-IPL thickness, visual acuity and visual hallucinations with disease severity in PD.

	Crude			Adjusted for age		
	B	95% CI	p-value	B	95% CI	p-value
GCL-IPL vs BCVA	0.011	0.003–0.20	0.01*	0.004	− 0.003–0.011	0.217
GCL-IPL vs MoCA	0.356	0.095–0.617	0.008*	0.246	0.004–0.487	0.046*
GCL-IPL vs mHY	− 0.04	− 0.07–− 0.01	0.021*	− 0.02	− 0.05–0.00	0.05
BCVA vs mHY	− 1.56	− 2.40–− 0.71	< 0.001*	− 1.18	− 2.10–− 0.26	0.012*
VH vs mHY	0.87	0.36–1.37	0.001*	0.69	0.18–1.21	0.01*

*GCL-IPL* combined ganglion cell layer and inner plexiform layer, *vs* versus, *BCVA* best-corrected visual acuity, *MoCA* Montreal cognitive assessment score, *mHY* modified Hoehn & Yahr stage, *VH* visual hallucinations, *B* regression coefficient, *CI* confidence interval.

*A p value p < 0.05 was considered statistically significant.

## Discussion

In this exploratory study, we demonstrated that the presence of VH in PD is associated with thinning of the GCL-IPL and, possibly, with a reduced BCVA. These observations suggest that ocular pathology may play a role in the development of VH in PD. In a previous study investigating the association of VH with the thickness of the various different retinal layers in PD, an association was found between the presence of VH and thinning of the RNFL, not with thinning of the GCL-IPL^15^. However, considering that the RNFL consists mainly of the axons of the cells forming the GCL, we believe that our present findings (an association between presence of VH and GCL-IPL thinning) are not in total contradiction with this study. Moreover, our findings are in line with histopathological research showing that PD pathology (i.e. degeneration of amacrine cells and abnormal alpha synuclein depositions) is mainly located in the GCL-IPL^10^. In light of the small sample size of the present study, we did not additionally investigate RNFL thickness, but chose to focus on the GCL-IPL only, since these retinal layers seem to be most interesting from a pathological point of view. Moreover, in comparison to the other retinal layers, the GCL-IPL is most strongly associated with visual dysfunction^10,17^.

In the present study, VH were associated with a reduced BCVA in PD, which is in line with previous research^1,18^. GCL-IPL measurements did not correlate with BCVA after correction for age, in agreement with previous studies, in which GCL-IPL thickness correlated with low contrast visual acuity only^17,19^, not with high contrast visual acuity (investigated in the present study). In contrast, in one other study, a correlation between retinal thickness and high contrast visual acuity was reported^20^, possibly due to the lack of a correction for age. Another potential explanation for this discrepancy is that our study may have lacked enough power to find such a correlation.

A lack of power may also have contributed to the absence of a significant difference in GCL-IPL measurements between the PD group and the control group in this study, which is in agreement with some studies^15,17,21,22^, yet in contrast with other studies^23,24^. This disparity could also be explained by methodological differences (i.e. OCT machine, segmentation software, analysis of one selected eye per patient versus the mean of both measured eyes per patient).

We observed that 35% of PD patients had visual hallucinations, which is in line with literature^1–3^. Furthermore, all outcome measures correlated with disease stage in PD (although for GCL-IPL thickness only a trend was found), suggesting that GCL-IPL thinning and a reduced visual acuity, as well as VH, may result from progression of disease-related pathology.

Moreover, our results seem to support the theory that dysfunction of both the peripheral visual system (i.e., impaired visual input) and central visual networks (i.e., impaired visual attention), contribute to the formation of VH in PD^5,6^. The association of VH with GCL-IPL thinning in our study highlights the potential role of retinal pathology in the formation of VH in PD, possibly through impaired visual input. In the present study, we could not support this contention by a correlation between GCL-IPL thinning and a reduced BCVA. However, this theory is supported by previous research showing an association between GCL-IPL thinning and specifically low contrast BCVA (not the high contrast BCVA we investigated)^17^. The role of the peripheral visual system in the development of VH in PD is further supported by the association of the presence of VH with reduced BCVA we observed. In addition, thinning of the GCL-IPL may be associated with an impairment of visual attention, as postulated by previous research^17^, which may further facilitate the development of VH in PD. This is supported by our observations of a correlation between GCL-IPL thinning and impaired cognitive function, as well as by the lower MoCA and CLOX1 score in patients with VH than in patients without VH. Taken together, GCL-IPL thinning may play a role in the development of VH by contributing to dysfunction of the peripheral visual system on the one hand, and to dysfunction of central visual networks on the other.

There are limitations to our study. First and foremost, the presence of VH was subjective to patients’ recall, which carries the risk of recall bias, especially in patients with cognitive deficits. We tried to reduce the risk of underestimating the true prevalence of VH by evaluating the patients’ medical records for additional information.

Second, all PD patients were on dopaminergic treatment, which may have triggered or worsened VH^25^. Therefore, the fact that patients with VH had a higher LED than patients without VH was not unexpected. However, levodopa is believed to have a protective effect on the retina and thus is not very likely to have caused GCL-IPL thinning in the patients with VH^26,27^. Moreover, we corrected for LED in the analysis of the association between the presence of VH and GCL-IPL thickness.

Lastly, considering the exploratory design of this study we could not establish any definitive causal relationships between the investigated variables. This would require a future longitudinal study in PD patients, combining repeated measurements of retinal layer thickness and parallel assessments of the presence of visual hallucinations over time, taking into account the severity of visual hallucinations. Ideally, this study should include a comprehensive evaluation of cognitive function, disease severity and dopaminergic medication use, and have sufficient power to correct for these confounders.

In conclusion, in patients with PD, visual hallucinations appear to be associated with a thinning of the inner retinal layers and, possibly, with reduced visual acuity. Further research using a longitudinal design is necessary to confirm these findings and to establish the causality of these relationships.

## Methods

### Study design and patients

This study involved data obtained in 40 PD patients and in 22 age- and sex-matched healthy controls, most of whom were included in a previously published study^28^, which was performed in the OLVG West (Amsterdam, the Netherlands) between July 1, 2017 and November 21, 2017. In summary, all included patients were diagnosed with PD by a neurologist specialized in movement disorders, fulfilled the UK Parkinson’s Disease Society Brain Bank Criteria, had a modified Hoehn and Yahr (mHY) stage between 2 and 5, had a disease duration of at least three years and were older than 50 years. Exclusion criteria for all participants were a neurodegenerative disorder other than PD, and a visual acuity of the best eye below 0.1. The study was performed in accordance with the Declaration of Helsinki Principles and the study protocol was approved by the Medical Ethics Committee of Amsterdam UMC, location VU University Medical Centre in Amsterdam, the Netherlands. All participants provided written informed consent.

For the present study, we included three patients that were excluded from our earlier study: two patients with strabismus and one patient with amblyopia. In these three cases, only the healthy eyes were included in the analysis. Furthermore, we excluded the eyes of participants with glaucoma or an intra-ocular pressure of 21 or higher (both eyes of three PD patients) and the eyes where the segmentation was unreliable due to insufficient OCT scan quality (both eyes of one PD patient and one healthy control). Patients were examined while they were in the “on” state.

### Neurological and ophthalmological assessments

All participants were examined by a neurologist specialized in movement disorders to establish the clinical diagnosis of PD in PD patients and to exclude the presence of a neurodegenerative disorder in controls. To evaluate cognitive function, The Montreal Cognitive Assessment (MoCA) and CLOX1 (an executive clock drawing test^29^) were used. Furthermore, the neurologist interviewed each participant for the presence of VH using standardized questions on minor hallucinations and complex visual hallucinations, within the last six months. Questions on minor hallucinations included questions on visual illusions (misperception of actual visual stimuli), presence hallucinations (feeling the presence of a person who is not really there) and passage hallucinations (hallucination of an object, animal or person passing through the peripheral visual field). In addition, questions on complex visual hallucinations included questions on hallucinations of objects, animals or persons and on the retainment or loss of insight. Furthermore, participants were asked to categorize the frequency in which they experienced VH into daily, weekly, monthly or never. While questioning the participants, the neurologist was also informed by the patients’ medical files. Presence of VH was defined as minor or complex visual hallucinations, occurring monthly or more frequently, in the last six months before inclusion.

After the neurological assessment, patients underwent an ophthalmologic examination, including measurement of best-corrected visual acuity (BCVA, with decimal notation, 1.0 equivalent to normal acuity), tonometry to measure intraocular pressure (IOP), slit lamp examination, funduscopy and optical coherence tomography (OCT) of the optic nerve and of the macula using a Spectralis OCT system (Heidelberg Engineering, Dossenheim, Germany; software version 6.0c with standard settings, including 3.2-mm macular scans centred on the fovea, with ART set at 100; subsequently, the scans were automatically segmented with software version 6.3.4.). For each macular scan, we obtained the GCL and IPL thickness for each quadrant separately.

### Statistical analysis

Statistical analysis was performed using IBM SPSS Statistics Version 23. Descriptive information is provided for the PD group and the healthy control group and for PD patients with and without VH separately. The primary outcome measure was the combined GCL and IPL (GCL-IPL) thickness in patients with VH compared to patients without VH, corrected for age, disease stage (mHY), levodopa use (LED) and cognitive function (MoCA), which was analysed using logistic GEE analysis. As the retinas of both eyes in a PD patient may be unequally affected^30^, we separately analysed both eyes in each patient and applied a correction for the duplication of retinal data and correlated observations in each patient, by making an adjustment in the standard errors with the dynamic GEE model. With this dynamic GEE model, we also made similar adjustments for including the thickness of all four quadrants of the GCL-IPL in the analysis.

Secondarily, we explored the association of VH with BCVA, corrected for age, disease stage, LED and cognitive function, and the correlation of the GCL-IPL thickness with BCVA and cognitive function (MoCA), using a correction for age (with logistic GEE analysis). In addition, we assessed the correlation of the outcome measures with disease stage (mHY), including the relationships between GCL-IPL thickness and mHY, BCVA and mHY (both analyses with linear GEE analysis), and VH and mHY (analysed with linear regression analysis), which all included a correction for age.

A p value of p < 0.05 was considered statistically significant. In view of the exploratory nature of this study, we decided not to correct for multiple testing^31^.

The datasets generated and/or analysed during the current study are available from the corresponding author on reasonable request.
